# Use of a therapeutic communication application in the Nursing undergraduate program: randomized clinical trial*

**DOI:** 10.1590/1518-8345.4461.3456

**Published:** 2021-06-28

**Authors:** Manuela de Mendonça Figueirêdo Coelho, Karla Corrêa Lima Miranda, Regina Claúdia de Oliveira Melo, Linicarla Fabiole de Souza Gomes, Ana Ruth Macêdo Monteiro, Thereza Maria Magalhães Moreira

**Affiliations:** 1 Universidade Federal do Ceará, Departamento de Enfermagem, Fortaleza, CE, Brazil.; 2 Universidade Federal do Ceará, Fortaleza, CE, Brazil.; 3 Centro Universitário Fametro, Fortaleza, CE, Brazil.

**Keywords:** Educational Technology, Randomized Controlled Trial, Higher Education, Nursing, Mobile Applications, Communication, Tecnologia Educacional, Ensaio Clínico Controlado Randomizado, Educação Superior, Enfermagem, Aplicativos Móveis, Comunicação, Tecnología Educativa, Ensayo Clínico Controlado Aleatorio, Educación Superior, Enfermería, Aplicaciones Móviles, Comunicación

## Abstract

**Objective:**

to analyze the effect on the knowledge of therapeutic communication by Nursing students through the use of applications.

**Method:**

a randomized and controlled clinical trial conducted with 60 nursing students. In the topic Groups and therapeutic communication techniques, the intervention-IG group (n=30) used the application and the control-CG group (n=30) was submitted to the traditional class. The pre- (Zero Test -0) and post-test knowledge (immediate-Test 1 and after 30 day-Test 2) of those involved were evaluated.

**Results:**

Test 1, performed immediately after the intervention, showed a mean of 11 hits in the control group and 13 in the intervention, with statistical significance (p=0.036). Test 2 showed a decrease in hits in both groups (IG=10.87 and CG=9.3), but maintained the difference between IG and CG in the post-test (p<0.01).

**Conclusion:**

the use of the application on therapeutic communication favored the knowledge of the students, when compared to the traditional teaching method. REBEC RBR-4TF6MR Registration.

## Abstract (pt)

**Objetivo:**

analisar o efeito no conhecimento sobre a comunicação terapêutica pelos acadêmicos de Enfermagem com o uso de aplicativos.

**Método:**

ensaio clínico randomizado e controlado realizado junto a 60 estudantes de Enfermagem. No tópico Grupamentos e técnicas de comunicação terapêutica, o grupo intervenção-GI (n=30) utilizou o aplicativo e o grupo controle-GC (n=30) foi submetido à aula tradicional. Foi avaliado o conhecimento pré (Teste zero -0) e pós-teste (imediato-Teste 1 e após 30 dias-Teste 2) dos envolvidos.

**Resultados:**

o teste 1, realizado imediatamente após a intervenção, mostrou uma média de 11 acertos no grupo controle e 13 no intervenção, com significância estatística (p=0,036). O teste 2 mostrou uma diminuição dos acertos nos dois grupos (GI=10,87 e GC=9,3), mas manteve a diferença entre GI e GC no pós-teste (p<0,01).

**Conclusão:**

a utilização do aplicativo sobre a comunicação terapêutica favoreceu o conhecimento dos estudantes, quando comparado ao método tradicional de ensino. Registro REBEC RBR-4TF6MR.

## Abstract (es)

**Objetivo:**

analizar el efecto del conocimiento sobre comunicación terapéutica por los académicos de enfermería con el uso de aplicativos.

**Método:**

ensayo clínico aleatorio controlado, realizado en 60 estudiantes de enfermería. En el tópico, agrupamientos y técnicas de comunicación terapéutica, el grupo intervención-GI (n=30) utilizó el aplicativo y el grupo control-GC (n=30) fue sometido a una clase tradicional. Fue evaluado el conocimiento pretest (Test cero -0) y postest (inmediato-Test 1 y después de 30 días-Test 2) de los participantes.

**Resultados:**

el test 1, realizado inmediatamente después de la intervención, mostró media de 11 aciertos en el grupo control y 13 en la intervención, con significación estadística de p=0,036. El test 2 mostró diminución de los aciertos en los dos grupos (GI=10,87 y GC=9,3), pero mantuvo diferencia entre GI y GC en el postest (p<0,01).

**Conclusión:**

la utilización del aplicativo sobre comunicación terapéutica favoreció el conocimiento de los estudiantes al ser comparado con el método tradicional de enseñanza. Registro REBEC RBR-4TF6MR.

## Introduction

The Nursing undergraduate courses aim to accompany changes in the health needs of society, the training process and health work. Integrated disciplines, active and student-centered methods have been a relevant challenge, instigating students and teachers to overcome the traditional method of teaching-learning^(^ 1 ^-^ 2 ^)^. A study predicts that soon nurses will experience their practice permeated with technology, requiring curricular adaptation^(^ 3 ^-^ 6 ^)^ that favors the early use of media resources in the undergraduate course and in care, as in the case of mobile devices or applications (APPs)^(^ 7 ^-^ 9 ^)^.

Although there is no guideline or legislation on the use of applications for teaching, the Brazilian Ministry of Education created the International Bank of Educational Objects, which provides virtual tools produced by teachers in free format, with multiple platform and language. But this bank has received few registrations, raising the need to increase its production^(^ 10 ^)^.

In this sense, review studies^(^ 11 ^-^ 12 ^)^ denote the adoption of multiple Learning Objects (OAs), such as blogs, chats, wikis and virtual simulations, in the increase of knowledge and autonomy of students, especially in the health area, suggesting their testing in clinical trials for the production of scientific evidence^(^ 12 ^)^.

When considering the complexity of nursing care, which requires the nurse, polysemy and completeness of content^(^ 13 ^)^, communication is the basic instrument of care, allowing centralization in the user and in their singularities^(^ 14 ^)^. Communication, whether verbal, non-verbal or para-verbal, is strongly present in the practice of the nurse, constituting action, including therapeutic, as the actions of nursing care provided to pregnant women, deaf people, mother-child binomial, critical patients, among others^(^ 15 ^-^ 18 ^)^.

The theoretical reference of communication adopted in this study was that of Maguida Costa Stefanelli, who considers the communication in Nursing as an integrator between assistance, teaching and research, being fundamental in the care, educational practice and health care^(^ 19 ^-^ 20 ^)^. Thus, the objective of this study was to analyze the effect on the knowledge of therapeutic communication by Nursing academics using applications

## Method

A parallel randomized clinical trial conducted from February to March 2017 with undergraduate Nursing students from a private faculty in the Fortaleza-Ceará educational network. Participants were randomly separated into two groups: Intervention Group (IG) (class using APP on therapeutic communication) and Control Group (CG) (traditional expository class on the same theme).

The intervention took place on a school day of the discipline “Psychological Basis for the Care Process”, in the class on “therapeutic communication”. In the curricular matrix of the course, this subject is taught in the third semester. The population of the study was 146 students enrolled in the discipline and the sample was 121 of these who were present in class during the data collection. The subject was taught in different shifts, minimizing the contact between participants.

Grouped randomization via www.random.org was used and the classes of the three shifts had the same chance to participate in the IG and the CG^(^ 21 ^)^, because the computerized algorithm defined which group would receive the intervention and which would be the control. Grouped randomization was chosen because individual randomization would not guarantee that students from the same class and different groups in the research would exchange information about the study.

In the institution researched, the Nursing course has existed for 15 years, currently with a maximum grade of five by the Ministry of Education (MEC), having already graduated more than two thousand nurses. It was found the absence of any discipline with systematic use of APP in teaching. It was inclusion criterion: to have smartphone with Android operating system compatible with the execution of APP (the tool was produced in Java programming language and, initially, only for such operating system). The exclusion criteria were defined as: previous failure in the subject; being under 18 years old and claiming not to have basic computer knowledge or use of technology. It was discontinuity criteria: no follow-up in post-test 1 (students who left the classroom before the end of the class) and/or post-test 2 (30 days after the intervention, two visits were made to the classroom, with 30-minute intervals between one visit and another, not finding the student).

To ensure blinding, the principal researcher (creator of APP) did not attend the class at IG and CG. The class, in both groups, was taught by an external researcher teacher, experienced, trained and experienced in teaching the subject, directed by a Standard Operating Procedure (POP). The POP detailed how the IG and CG class should be, containing the educational objective and the detailed description of the approach and the activities to be carried out by the teacher.

The difference between the IG and the CG was in the discussion about groupings and therapeutic communication strategies, carried out in the CG in an expository-dialogue way and mediated by PowerPoint slides, and in the IG with the handling and exploration of the APP. The same researcher, who taught the class at IG and CG, applied the data collection questionnaire in all three stages. Blinding in educational interventions is complex and needs to minimize possible co-interventions and blind, minimally, the responsible for the evaluation and adjudication of the outcomes, diminishing biases of the principal researcher in a differentiated look to the IG^(^ 21 ^)^.

The IG and CG classes began with the conceptual presentation of human communication, its forms and functions, therapeutic relationship, groupings (expression, clarification and validation)^(^ 19 ^)^ and strategies of therapeutic communication (silence, listen reflexively, verbalize acceptance, verbalize interest, use open sentences, repeat user comments, ask questions, return the question asked, use descriptive phrases, allow the user to choose the subject, focus on the main idea, verbalize doubts, say no, stimulate the expression of feelings, use humor therapeutically, stimulate comparisons, request clarification of unusual terms, identify the agent of the action, describe events in logical sequence, repeat the user’s message, request the user to repeat what was said, summarize the content of the interaction)^(^ 19 ^)^.

The CG class was taught in the morning group and held exposition with the PowerPoint feature and duration of one hour and 47 minutes. In the night group (IG), following the POP, in the topic Groups and therapeutic communication strategies, the APP “Therapeutic Communication” was used. For this, students were guided to download the APP in temporary link using a specific login and password. The APP resources were handled, which also contained an interactive video about the practice of content. This class lasted one hour and 52 minutes. They were told to feel free to explore the APP, watch and interact with the video and that they could dialogue through the teacher’s exposure and the content of the APP. After the class, students’ access to APP was cancelled to prevent other students from accessing it. It should be noted that APP was produced and validated for this essay, and this methodological study is under evaluation in a journal for later publication.

The APP presents each therapeutic communication strategy, as well as its referred grouping, with clinical examples and a video with a simulation of the Nursing consultation in which the student can identify the various strategies used, thus being able, in addition to obtaining a theoretical contextualization, to identify, in simulation, the use of the techniques during clinical practice.

Before the IG and CG class (Moment 0/M0), the Learning Verification Test (TVA) was applied to the students, which was reapplied immediately after the end of the class (Moment 1/M1) and 30 days after (Moment 2/M2). The follow-up, after 30 days of class, was given to compare immediate and long-term learning.

The TVA was built by researchers based on a literature review on the subject and validated by seven expert judges. With a maximum score of up to twenty points, it contains questions of sociodemographic characterization [age (self-declared in years), gender, marital status and occupation] and 20 open questions worth one point each on the concept of communication, practices with grouping and situations to identify the use of therapeutic communication techniques.

The outcome was the learning (acquired knowledge) defined as the ability of the participants to identify groups of therapeutic communication, associate their respective strategies and use them in specific clinical situations in M0, M1 and M2. The average time to fill the TVA was nine minutes.

The data was processed in the Statistical Package for the Social Sciences (SPSS), version 22.0, and presented in graphs and tables. After verifying data normality with the Kolmogorov-Smirnov test for independent samples, Student’s t test was used to compare means and Fisher’s exact test. Analysis of variance (one-way ANOVA) was used for intergroups with Sidak post-hoc analysis. The 95% confidence interval and statistical significance were considered when p <0.05. The study was approved by the Ethics Committee of the State University of Ceará (UECE), with the Ordinance No. 1.570.217, and registered in the Clinical Trials Registry platform (REBEC) with the number RBR-4TF6MR.

## Results

**Figure 1:**
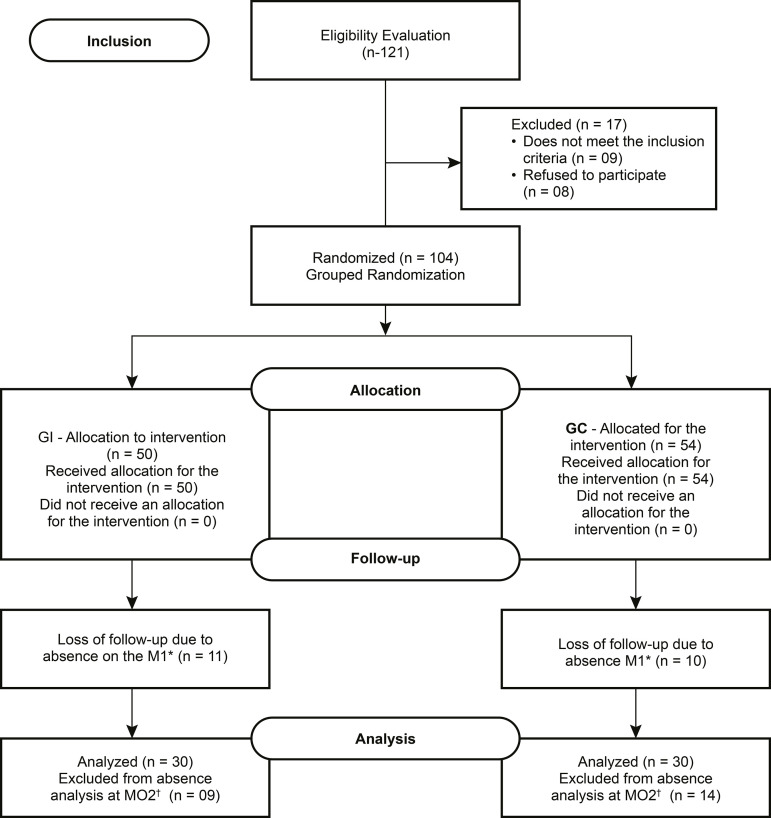
CONSORT diagram. Fortaleza, Ceará, Brazil, 2017 *Moment 01; ^†^Moment 0

Of the 121 students invited, 104 met the inclusion criteria and accepted to participate in the study, 50 were assigned to IG and 54 to CG. During the research, the IG had 11 losses for absence in the M1 and another nine for absence in the M2, ending with 30 students. In the CG, there were ten losses for absence at the M1 and 14 more for absence at M2, ending also with 30 participants (Figure 1). The recruitment and follow-up took place in February and March of 2017.

The mean age was 24.4 years (SD=3.8) in IG and 21.3 (SD=4.2) in CG, where age homogeneity was confirmed (p=0.301). Occupation (p=0.873), marital status (p=0.651) and gender (p=0.582) were also homogeneous between groups (Table 1).

**Table 1:** Characterization of the participants of the Intervention Group (IG) and Control Group (CG). Fortaleza, Ceará, Brazil, 2017

Variables	Intervention Group IG		Control Group CG		p*
**Work**	**N**	**%**	**n**	**%**	
Yes	12	60.0	08	40.0	**.873**
No	18	35.0	22	55.0	
**Marital Status**					**.651**
Married	10	60.0	08	40.0	
Not Married	20	35.0	22	55.0	
**Sex**					
Female	28	54.9	23	45.0	**.582**
Male	02	22.3	07	77.7	

* Fisher's exact test

The average of inter-group hits was similar at M0, being 7.37 at IG and 7.27 at CG, without statistical significance, announcing the homogeneity in the previous knowledge of the content between the two groups (Table 2).

 

**Table 2:** Mean of hits, standard deviation and p-value of the mean of IG and CG at Moments M0, M1 and M2. Fortaleza, Ceará, Brazil, 2017

	Pre-test			Post-test 1 (Immediate)			Post-test 2 (30 days)		
	Average of hits	SD^*^	p^†^	Average of hits	SD^*^	p^†^	Average of hits	SD^*^	p^†^
**IG**	7.37	2.84		13	2.80		10.87	2.5	
**CG**	7.27	2.27	**.945**	11	2.10	**.036**	9.3	2.7	**0.01**

* Standard Deviation;

† Valor of p obtained from Student's t test

In the M1, the IG had an average of 13 hits and the CG had 11 hits, with statistical significance between groups (p=0.036). In M2, the number of hits of both groups decreased (IG = - 2.13 + 2.5 and CG = - 1.7+2.7), but the difference between IG and CG remained, with statistical significance (p=0.01). Moreover, in IG, the decrease of the mean hits was numerically smaller than the standard deviation and larger than the CG, although the standard deviation decreased in IG and increased in CG, when comparing [M1 - (minus) M2] to [M1 - (minus) M0], showing greater homogeneity in the hits of students in IG.

Even with more students decreasing their grades in M2, still the students in IG showed higher values than those in CG, with statistical significance (p=0.028) and greater homogeneity of values (Table 3). The study was finalized after data collection from M2.

**Table 3:** Number of hits and difference *per* IG and CG participant at M0, M1 and M2. Fortaleza, Ceará, Brazil, 2017

Student (S)	Number of hits and difference between the moments (M0-M1-M2) of the Intervention Group (IG)					Number of hits and difference between the moments (M0-M1-M2) of the Control Group (CG)				
	M0	M1	M1-M0	M2	M2-M0	M0	M1	M1-M0	M2	M2-M0
S01	11	15	+04	13	+02	08	14	+04	10	+02
S02	03	12	+09	10	+07	06	10	+04	07	+01
S03	05	09	+04	10	+05	02	10	+08	11	+09
S04	09	11	+02	11	+02	07	09	+02	08	+01
S05	12	17	+05	15	+03	06	12	+06	12	+06
S06	07	14	+07	13	+06	07	08	+01	08	+01
S07	06	13	+07	12	+06	11	11	00	13	+02
S08	09	17	+08	15	+06	05	15	+10	07	+02
S09	04	08	+04	07	+03	06	12	+06	04	+02
S10	09	15	+06	14	+05	10	11	+01	10	00
S11	07	15	+08	11	+04	11	11	00	11	00
S12	10	12	+02	09	-01	12	15	+03	15	+03
S13	08	15	+07	13	+05	07	10	+03	09	+02
S14	04	09	+05	08	+04	07	11	+04	09	+02
S15	13	17	+04	14	+01	06	10	+04	09	+03
S16	06	12	+06	10	+04	09	10	+01	08	-01
S17	14	17	+03	15	+01	06	10	+04	05	-01
S18	04	10	+06	07	+03	06	12	+06	09	+03
S19	10	16	+06	12	+02	09	13	+04	11	+02
S20	05	11	+06	09	+04	12	12	00	14	+02
S21	09	17	+08	14	+05	06	15	+09	14	+08
S22	08	17	+09	12	+04	07	12	+05	10	+03
S23	05	12	+07	07	+02	08	09	+01	05	+03
S24	06	11	+05	09	+03	05	09	+04	06	+01
S25	08	14	+06	11	+03	07	08	+01	11	+04
S26	04	15	+11	10	+06	06	13	+07	07	+01
S27	07	10	+03	09	+02	09	10	+01	09	00
S28	05	12	+07	07	+02	05	07	+02	07	+02
S29	05	10	+05	09	+04	06	12	+06	10	+04

Note: students with the grey colored cell had increased knowledge

Both IG and CG showed more hits on M1 and M2 than on M0. In IG, the difference in the learning average [M1 (immediate)-M2 (30 days)] had an effect on learning within 30 days, although in many cases lower at M2 than at M1. In the case of CG, some students decreased or returned to the value obtained in M0.

The ANOVA test showed the effect of the intervention on learning by students from IG (p < 0.001) and CG (p < 0.001). Sidak’s *post-hoc* showed a significant difference between all moments in both groups (intragroup).

**Figure 2:**
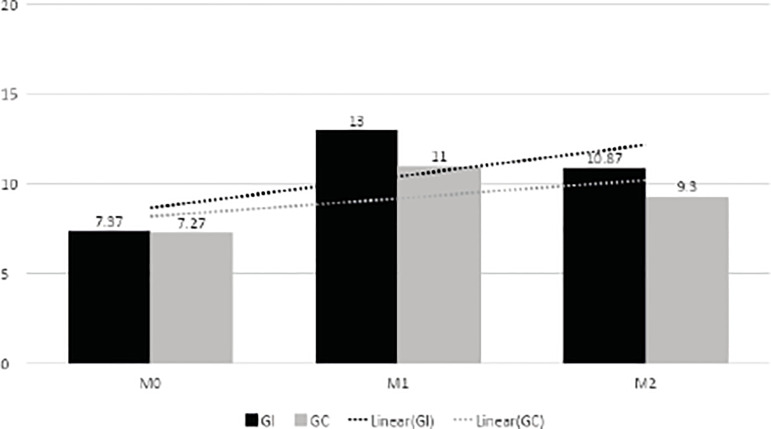
IG and CG average hits at Moments M0, M1 and M2 with their trend lines. Fortaleza, Ceará, Brazil, 2017

The following Figure 2 compares the averages of hits of the two groups at the three moments, associated with the trend lines.

## Discussion

At first, it should be considered that in experimental research, sampling bias is a significant problem. Thus, getting homogeneous samples, even unrepresentative ones, is one of the ways to minimize this issue^(^ 21 ^)^. The similarity in the study groups with the two groups is a way to ensure that they have the same characteristics, configuring the intervention as the only different variable to permeate the study^(^ 22 ^)^.

The on-screen study reveals that the average grades of the two moments after the intervention (M1 and M2) were higher in IG. A research carried out in Fortaleza presented an increased average of hits after educational intervention with APP, denoting the positive influence of this technology in the knowledge of students^(^ 23 ^)^. Similarly, in this study, the APP on the therapeutic communication in commentary had an effect on the learning of students in Nursing, which can have repercussions on the therapeutic communication of Nursing^(^ 24 ^)^. At Washington State University, research on the use of APP in teaching-learning was conducted and it was proven that the use of APP by students contributes to learning (p=0.01)^(^ 25 ^)^.

Therapeutic communication needs to be further explored by nurses in teaching and interactions during care. Communication strategies are powerful tools, but underused in the professional exercise of Nursing^(^ 26 ^)^, prompting reflections on communicational instrumentation during professional training. But it is worth mentioning that there may be confusion between the therapeutic communication strategies and the subjective issues of the professional, making affection, solidarity and compassion, among other feelings, be referred to as communication strategies. However, therapeutic communication techniques have specific groupings and are endowed with goals and indications for their use^(^ 27 ^)^, which should facilitate its application in the clinical practice of Nursing.

Therapeutic communication can be developed, that is, it is not necessarily a “born” constituent of the professional. Thus, since communication permeates the nurse-patient therapeutic relationship, it also requires study, nurse’s skill and applicability in the daily life of this area. This need can be met, in part, with the study via APP, which has already been verified, even in intervention in a shorter time (15 days)^(^ 28 ^)^.

A Peruvian clinical trial measured the knowledge acquired by nursing students after using APP to teach tracheostomy tube aspiration and pointed out that the knowledge of students who used APP was superior and of statistical significance (p=0.003) to the traditional one, recommending the adoption of such a device^(^ 29 ^)^.

A meta-analysis of 11 clinical trials showed the increase in learning via APP for nurses and students, stating that there is good support and positive influence on Nursing education^(^ 30 ^)^. However, using smartphones during the lesson can also be a distraction^(^ 31 ^)^, which can make it difficult to reach the intended goal.

The limitations of this study are the follow-up of patients for only 30 days and the use of the APP only once, in a single day, due to the need to finish the research. But the literature reinforces the need for therapeutic communication in professional exercise^(^ 24 ^,^ 26 ^-^ 27), although there is no description of how to do it. Thus, it has been proven that the application on therapeutic communication is conducive to teaching, being useful in situations of social withdrawal such as the one faced today.

## Conclusion

The use of APP for group teaching and therapeutic communication strategies has shown an effect on the knowledge of students who have used the technology, as opposed to the traditional model. The knowledge remained greater in the intervention group, even after thirty days of use of APP, configuring itself into a powerful teaching tool.

This study presents important contributions to teaching in Nursing, demonstrating that APP can facilitate contemporary teaching and learning, thus calling on professionals to develop, validate and use tools for teaching. It is recommended, in the future, to verify the repercussions of the use of APP in clinical practice on other health matters and with larger samples and follow-up deadlines.

## References

[1] Baldoino AS, Veras RM (2016). Analysis of Service-learning activities adopted in health courses
of Federal University of Bahia. Rev Esc Enferm USP.

[2] Lana LD, Birner AJ (2015). Case report on the construction and preparation of a portfolio as
a learning assesment method. Cienc Enferm.

[3] Risling T (2017). Educating the nurses of 2025: Technology trends of the next
decade. Nurse Educ Pract..

[4] Oliveira LL, Mendes IC, Balsells MMD, Bernardo EBR, Castro RCMB, Barbos APS (2019). Educational hypermedia in nursing assistance at birth: building
and validation of content and appearance. Rev Bras Enferm..

[5] Mota NP, Vieira CMA, Nascimento MNR, Bezerras AM, Quirino GS, Félix NDC (2019). Mobile application for the teaching of the International
Classification for Nursing Practice. Rev Bras Enferm..

[6] Serafim ARRM, Silva ANS, Alcântara CM, Queiroz MVO (2019). Construction of serious games for adolescents with type 1
diabetes mellitus. Acta Paul Enferm.

[7] Mackay BJ, Anderson J, Harding T (2017). Mobile technology in clinical teaching. Nurse Educ Pract.

[8] Alvarez AG, Sasso GTMD, Iyengar MS (2017). Persuasive technology in the teaching acute pain assessment in
nursing: results in learning based on pre and post-testing. Nurse Educ Pract.

[9] Salum NB, Junkes C, Amante LN, Mendez CML (2019). Mobile educational follow-up application for patients with
peripheral arterial disease. Rev. Latino-Am. Enfermagem.

[10] Bardy LR, Hayashi MCPI, Schlunzen ETM, Seabra MO (2013). Objects for Learning as educational resources in inclusive
contexts: support for distance teacher education. Rev Bras Educ Esp..

[11] Trindade CS, Dahmer A, Reppold CT (2014). Learning Objects: An Integrative Review in
Healthcare. J. Health Inform..

[12] Coelho MMF, Miranda KCL (2016). Learning objects used in nursing students training: integration
review. Rev Tend Enferm Profis..

[13] Fernandes JD, Rebouças LC (2013). A decade of National Curriculum Guidelines for Graduation in
Nursing: advances and challenges. Rev Bras Enferm.

[14] Amorim CB, Barlem ELD, Mattos LM, Costa CFS, Oliveira SG (2019). Disclosure of difficult news in primary health care: aspects that
hinder or facilitate communication from the perceptions of
nurses. Rev Gaucha Enferm.

[15] Beserra GL, Oliveira PMP, Pagliuca LMF, Almeida PC, Anjos SJSB, Pinheiro AKB (2019). Non-verbal nurse-parturient communication in labor in
Portuguese-speaking countries. Rev. Latino-Am. Enfermagem.

[16] Sanches ICB, Bispo LP, Santos CHS, França LS, Vieira SNS (2019). The role of the nurse in relation to the deaf
patient. Rev Enferm UFPE On Line.

[17] Teixeira TRF, Avila MAG, Braga EM (2019). Patients' understanding of nursing instructions in cardiac
catheterism: a qualitative study. Cogitare Enferm.

[18] Costa AR, Nobre CMG, Gomes GC, Rosa GSM, Nornberg PKO, Medeiros SP (2018). Perception of the family in a pediatric unit about nursing
care. Rev Enferm UFPE On Line.

[19] Stefanelli MC, Stefanelli MC, Carvalho EC (2005). Estratégias de comunicação terapêutica. A comunicação nos diferentes contextos da enfermagem.

[20] Stefanelli MC (1987). Teaching communication therapeutic techniques in nurse-patient
relationship: conceptual basis - Part II. Rev Esc Enferm.

[21] Hulley SB, Cummings SR, Brownwe WS, Grady DG, Neman TB (2015). Delineando a pesquisa clínica.

[22] Kara-Júnior N (2014). Definition of population and randomization of sample in clinic
surveys. Rev Bras Oftalmol.

[23] Pereira FGF, Caetano JÁ, Frota NM, Silma MG (2016). Use of digital applications in the medicament calculation
education for nursing. Invest Educ Enferm.

[24] Dermani DB, Garbuio DC, Carvalho EC (2020). Knowledge, applicability and importance attributed by nursing
undergraduates to communicative strategies. Rev Bras Enferm.

[25] Keegan RD, Oliver MC, Stanfill TJ, Stevens KV, Brown GR, Ebinger M (2016). Use of a Mobile Device Simulation as a Preclass Active Learning
Exercise. J Nurs Educ.

[26] Torres GMC, Figueiredo IDT, Cândido JAB, Pinto AGA, Morais APP, Araújo MFM (2017). Therapeutic communication in the interaction between health
workers and hypertensive patients in the family health
strategy. Rev Gaúcha Enferm.

[27] Almeida KLS, Garcia DM (2015). Use of communication strategies in palliative care in Brazil:
integrative review. Cogitare Enferm.

[28] Chuang YH, Lai FC, Chang CC, Wan HT (2018). Effects of a skill demonstration video delivered by smartphone on
facilitating nursing students' skill competencies and self-confidence: A
randomized controlled trial study. Nurse Educ Today.

[29] Bayram SB, Caliskan N (2019). Effect of a game-based virtual reality phone application on
tracheostomy care education for nursing students: A randomized controlled
trial. Nurse Educ Today.

[30] Kim JH, Park H (2019). Effects of Smartphone-Based Mobile Learning in Nursing Education:
A Systematic Review and Meta-analysis. Asian Nurs Res (Korean Soc Nurs Sci).

[31] Zarandona J, Cariñanos-Ayala S, Cristóbal-Domíngues E, Martín-Bezos J, Yoldi-Mitxelena, Cillero IH (2019). With a smartphone in one's pocket: A descriptive cross-sectional
study on smartphone use, distraction and restriction policies in nursing
students. Nurse Educ Today.

